# Antifungal susceptibility and phenotypic characterization of oral isolates of a black fungus from a nasopharyngeal carcinoma patient under radiotherapy

**DOI:** 10.1186/s12903-015-0023-9

**Published:** 2015-03-20

**Authors:** Chaminda Jayampath Seneviratne, Phoenix HL Fong, Sarah SW Wong, Victor HF Lee

**Affiliations:** Oral Sciences, Faculty of Dentistry, National University of Singapore, 11 Lower Kent Ridge Road, Singapore, 119083 Singapore; Oral Biosciences, Faculty of Dentistry, The University of Hong Kong, Pok Fu Lam, Hong Kong; Department of Clinical Oncology, Li Ka Shing Faculty of Medicine, Queen Mary Hospital, The University of Hong Kong, 1/F, Professorial Block, Queen Mary Hospital, Pok Fu lam, Hong Kong

**Keywords:** Black fungus, Antifungal susceptibility, Biofilm, Virulence

## Abstract

**Background:**

During a research project on fungal *Candida* species in patients wearing obturator treated with radiotherapy for their recurrent nasopharyngeal carcinoma, we serendipitously observed the presence of black fungus in two consecutive samples from a patient.

**Case presentation:**

The samples were collected from a 57 year-old Hong Kong gentleman who diagnosed to have undifferentiated type of nasopharyngeal carcinoma. He was treated with definitive concurrent chemoradiotherapy followed by adjuvant chemotherapy and then received a second-course radiotherapy with IMRT. 18S rDNA sequencing revealed that the isolates belong to *Exophiala dermatitidis* which was susceptible to fluconazole, itraconazole, ketoconazole and voriconazole. Interestingly, *E. dermatitidis* isolates were resistant to caspofungin and one isolate was resistant to amphotericin B. Both isolates formed biofilms comparable to that of *Candida albicans*. Single isolate of *E. dermatitidis* showed hemolysin and proteinase ability comparable to *C. albicans* whilst the other isolate was not.

**Conclusion:**

We, for the first time, reported the discovery of a black fungus–*E. dermatitidis* isolates derived from a patient with nasopharyngeal carcinoma treated with radiotherapy. These isolates were shown to be resistant to caspofungin, a major antifungal agent for systemic candidiasis. As little is known about the black fungus in the clinical setting, it is important that clinicians must keep abreast of the new discovery in this field.

## Background

Oral fungal infections are not common in normal healthy humans. However, opportunistic mycoses may occur in immunosuppressed individuals, for instance, patients with HIV infection and AIDS, and cancer patients receiving chemotherapy and/or radiotherapy [1,2]. The incidence of nasopharyngeal carcinoma (NPC) is rare in most parts of the world, but it is endemic among Asia, especially in Southern China including Hong Kong [3]. Radiotherapy has been the standard treatment for early-stage NPC while radiotherapy combined with chemotherapy is reserved for locally and regional advanced non-metastatic diseases [4]. The major oral health adversary of NPC patients is the radiation effect on salivary glands, resulting in salivary dysfunction, xerostomia and oral mucositis which played a significant role in oral cavity infection and contributed to *Candida* infection [5,6]. Significant portions of the parotid glands and oral cavity inevitably suffer from high dose of radiation, owing to the close proximity of these structures to the tumour. It has been reported that 50 - 80% of patients developed xerostomia during intensity modulated radiation therapy (IMRT), reaching to almost 100% when concurrent chemotherapy is given [5]. Saliva plays a major role in oral homeostasis, modulating the health of the oral cavity. Salivary immunoglobulins are important to the oral defense, which protect mucosal tissues from microbial infection [7]. Therefore, these patients are particularly prone to oral fungal infections. *Candida* and *Aspergillus* are the two most important opportunistic fungal species [1]. Fungal diseases caused by other species like *Coccidiodes*, *Histoplasma* are exceedingly rare.

*Exophiala dermatitidis* is a member which belongs to the fungal order Chaetothyriales, which can cause infection in both healthy and immunocompromised individuals [8]. Chaetothyriales are the principal causative agents of human phaeohyphomycosis and other types of clinical patterns like chromoblastomycosis [8,9]. *E. dermatitidis* have been found worldwide in human-made environment, for example the bathing facilities in Asia and the public Turkish steam bath facilities in Europe [10,11]. It has been proven oligotrophic and thermophilic, and is able to survive in hot and moist conditions [8].

It should be noted that *E. dermatitidis* is regarded as an emerging systemic pathogen in Southeast Asia [8]. Fatal cerebral and disseminated black fungi infections have been reported in China, and *E. dermatitidis* was one of the causative agents [12]. Previously, *E. dermatitidis* has never been reported to be isolated from the oral cavity in humans. In this case report, two strains of *E. dermatitidis* were isolated from an obturator of a patient with NPC during IMRT. We comprehensively performed a molecular analysis of the isolates and characterized their phenotypic behaviours, in terms of antifungal susceptibility, hemolysin and proteinase production and biofilm formation.

## Case presentation

### Case

A 57 year-old Hong Kong gentleman was diagnosed to have undifferentiated type of nasopharyngeal carcinoma (NPC) (stage III T2N2M0 disease, AJCC 7th edition) at Queen Marry Hospital, Hong Kong in March 2008. He was treated with definitive concurrent chemoradiotherapy followed by adjuvant chemotherapy completed in September 2008. He was found to have local recurrence of NPC in the nasal cavity extending to the right ethmoid and maxillary sinus in August 2012. Craniofacial resection and right maxillectomy was performed in October 2012. Pathology report showed recurrent undifferentiated carcinoma. An oral obturator was custom-made for filling up the wound defect and facilitation of mastication, swallowing and oral diet. In view of positive resection margin, post-operative chemoradiotherapy was suggested. He then received a second-course radiotherapy with IMRT with 60Gy delivered to the operative bed and 56Gy to the high risk region, all in 30 fractions over 6 weeks by simultaneous accelerated radiation therapy (SMART) technique concurrent with 2 cycles of intravenous chemotherapy with cisplatin 100 mg/m^2^ every 3 weeks.

### Materials and methods

#### Mouth rinse samples

This patient was invited for a study conducted at Department of Clinical Oncology, Queen Marry Hospital, The University of Hong Kong, Hong Kong aiming at the identification of the host and pathogen attributes of oral candidiasis in patients with NPC treated with IMRT. Written informed consent was obtained from the patient to take part in the study. Approval for this study in compliance with Helsinki Declaration from local institutional review board of the University of Hong Kong was obtained prior to study commencement. The patient was requested to rinse the mouth with 10 ml phosphate buffered saline (pH 7.3, 0.1 M) for 1 min and expectorate into a sterile container. Samples were then transferred to the Oral Biosciences Laboratory, Faculty of Dentistry, The University of Hong Kong for microbiological analysis. Patient was asked to expectorate stimulated saliva at baseline before the start of IMRT and then at 2, 4, 6 and 8 weeks after the commencement of IMRT. Mouth rinse samples were first centrifuged at 13,200 rpm for 10 minutes and the pellet was resuspended in sterile PBS followed by vortexing for 30 seconds. Thereafter, samples were plated on to a Sabouraud dextrose agar (SDA, Gibco). The plates were incubated for 48 hours at 37°C.

#### Speciation of the fungal isolates

The fungus was subcultured on SDA to obtain single colonies, which were then subjected to Gram stain and plated on CHORMagar for speciation. Next, isolates were subjected to commercially available API 32C identification system (BioMe’rieux, Marcy l’Etoile, France).

#### Molecular identification

In order to make precise identification, DNA of the black fungal samples was extracted using the MasterPure™ yeast DNA purification kit (Epicentre Biotechnologies, Madison, Wisconsin, US) according to the instructions of the manufacturer. The extracted DNA samples were amplified by PCR with fungus specific universal primers ITS3 and ITS4. The PCR amplification protocol consisted of 30 cycles of denaturation for 1 minute at 94°C, annealing for 1 minute at 50°C, and extension for 2 minute at 72°C. Amplicons were verified by electrophoresis on agarose gels staining with ethidium bromide. Next, amplicon was purified and subjected to 18S rDNA sequencing at Centre for Genome Research, The University of Hong Kong. DNA sequence was searched against NCBI database using standard criteria for a significant match.

#### Antifungal susceptibility testing

The antifungal susceptibility of the isolates was evaluated using the disc diffusion assay following the CLSI M44-A guideline with certain modifications as we have previously described [13,14]. Five commercially available antifungal agents were selected for this study: amphotericin B (AMPH), caspofungin (CASP5), fluconazole (FLUCZ), ketoconazole (KTC), itraconazole (ITRAC), and voriconazole (VOR.1) (Neo-Sensitabs, Rosco Diagnostica, Taastrup, Denmark). Suspensions equal to McFarland 0.5 turbidity from pure culture were prepared. Twenty microliters of the suspension were inoculated on Mueller-Hinton agar by spiral plating machine to achieve an evenly disturbed inoculation. Ten-microgram amphotericin B, 5-μg caspofungin, 25-μg fluconazole, 10-μg itraconazole, 15-μg ketoconazole and 1-μg voriconazole disks were then applied to the inoculated agar. Plates were incubated aerobically at 37°C for 2 days. Then the diameters of the growth inhibition zones were measured. The assay was performed in duplicates on two separate occasions.

#### Biofilm formation evaluated by XTT reduction assay

*E. dermatitidis* biofilms were developed according to a previously published protocol for fungal biofilms [15,16]. In brief, broth culture of *E. dermatitidis* was prepared by inoculating a loopful of culture into yeast nitrogen base (YNB, Difco) medium supplemented with 50 mM glucose for overnight incubation at 37°C. The culture was washed twice with 20 ml of phosphate-buffered saline (PBS; pH 7.2, 0.1 M) by centrifugation. The washed culture was resuspended in YNB medium supplemented with 100 mM glucose and obtained a suspension equal to McFarland 4 turbidity. One hundred microliters of the suspension were added into each well of a sterile 96-well polystyrene microtiter plate (Iwaki, Tokyo, Japan). One row of wells containing only the medium without any cell suspension was prepared as negative control. The plate was incubated for 1.5 hours at 37°C in a shaker at 75 rpm to allow the cells to adhere to the well surface (adherence phase). Then the cell suspension in each well was aspirated and washed with 100 ml of PBS to remove nonadherent cells. Two hundred microliters of YNB medium with 100 mM glucose was added to each of the washed wells, and the plate was incubated at 37°C in a shaker at 75 rpm for 48 hours.

The biofilm formation was evaluated by using an XTT reduction assay [15,17]. A mixture of 40 ml XTT (Sigma, St. Louis, MO) solution (1 mg/ml in PBS), 2 ml menadione (Sigma) solution (0.4 mM) and 158 ml PBS was prepared. After the 48-hour growth phase, all the cell suspensions were aspirated and washed with 200 ml of PBS for 3 times. Two hundred microliters of PBS-XTT-menadione solution were added to each of the washed wells, and the plate was incubated at 37°C in dark for 3 hours. Following the incubation, 100 ml of solution from each well was transferred to a new well and measured with a microtiter plate reader (SpectraMAX 340 Tunable Microplate Reader; Molecular Devices Ltd., Sunnyvale, CA) at 490 nm.

#### Hemolysin assay

Hemolytic activity was determined with the blood plate assay as we have previously described for other fungal species [18]. In brief, suspensions with an inoculum size of 10^8^ cells/mL of pure culture were prepared. Ten microliters of the suspension were spotted on Sabouraud dextrose agar supplemented with 3% glucose and 7% fresh sheep blood (wt/vol; Merck, Darmstadt, Germany). Plates were incubated at 37°C in 5% CO_2_ for 48 hours. The distinctive translucent halo around the inoculum site showed positive hemolytic activity, which was measured by using computerised image analysis system (Qwin, Leica, UK). The intensity of the hemolysin production by the fungal species was represented by hemolytic index (Hi), the ratio obtained by dividing the diameter of the colony by the total diameter of the colony and the translucent halo. *C. albicans* ATCC 90028 and *C. parapsilosis* ATCC 22019 strains were used as positive and negative control of hemolytic activity respectively. The assay was performed in quadruplicate on two separate occasions.

#### Proteinase assay

Bovine serum albumin (BSA) assay was performed for evaluating the proteinase activity as we have previously described [19]. Suspensions equal to McFarland 2 turbidity from pure culture were prepared. Ten microliters of the suspension were spot inoculated to BSA 1% (w/v) agar plate. Plates were incubated aerobically at 37°C for 5 days followed by stained with naphthalene black 1.25% solution in methanol/water 90% v/v for 15 minutes and decolorized for a further 36 hours with changing the solution. The diameter of the zone of proteolysis and the colony were measured by using computerised image analysis system (Qwin, Leica, UK). Proteinase production (Pr_d_ value) was obtained by dividing the diameter of the zone of proteolysis and the colony by the diameter of the colony. *C. albicans* ATCC 90028 and *C. parapsilosis* ATCC 22019 strains were used as positive and negative control of proteinase activity respectively. The assay was performed in quadruplicate on two separate occasions.

### Results and discussion

Although *Exophiala* are environmental fungi, its presence in clinical specimens collected in two consecutive visits, should not be disregarded as a contamination [20]. Black fungi have been known for decades, however they are among the most difficult fungal groups to identify, and therefore the diagnostic confusion was common in the past [8]. Due to the advancement of molecular techniques and availability of DNA sequences of different gene loci in sequence databases, such as GenBank, identification of *Exophiala* to the species level has been made possible. Previously, Hong Kong has reported only a single case of *E. dermatitidis* associated with acute myeloid leukemia 43 year-old female patient undergoing peripheral blood stem cell transplant [20]. However, it was isolated from stool samples.

**Figure 1:**
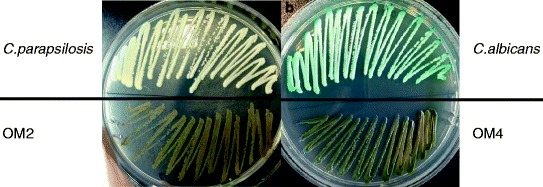
**Speciation of isolates by CHROMagar.** Pure culture of *C. parapsilosis* and OM2 were streaked on the same CHROMagar plate **(a)**. And pure culture of *C. albicans* and OM4 were streaked on another CHROMagar plate **(b)**. Both of OM2 and OM4 showed dark brown color on the plate, which were retaining their dark color. Then the colors of the cultures were observed and pictures were taken after 48-hour incubation.

Initially, we were looking to recover *Candida* species from this patient’s samples. However, we observed the appearance of unusual dark colonies on the SDA plates. The darkness of the color increased when the culture is getting older and after couple of days appeared as a “black fungus”. In order to obtain pure culture, the black fungal colonies were further subcultured on SDA for the second round, which were then subjected to Gram stain. Two black fungi isolates were found in two of mouthwash samples respectively, they were named as OM2 and OM4. OM2 and OM4 showed Gram positive cells under the light microscope, 1000× magnification, whose size, shape and color are similar to *C. albicans* with some hyphal elements. Next, we plated the isolate on CHORMagar alongside *Candida albicans* and *Candida parapsilosis*. Fungal isolates retained their black color on CHROMagar whilst *C. albicans* and *C. parapsilosis* showed classical green and white color respectively (Figure 1). We also employed classical *Candida* identification API 32C AUX method which showed negative results.

Referring to results from the disk diffusion assay, both of the black fungi isolates were resistant to caspofungin (Table 1). Moreover, it is noteworthy that OM2 isolate was also resistant to amphotericin B while OM4 isolate had intermediate susceptibility. Amphotericin B is regarded as the “gold standard” in the treatment of fungal infections [21]. Therefore, fungal strains that are resistance to amphotericin B should be given especial attention. However, both isolates were susceptible to the azole class of antifungals *viz.* fluconazole, ketoconazole, itraconazole, and voriconazole. Antifungal susceptibility data on the clinical *E. dermatitidis* isolates are still sparse despite the advances in testing methods. In general, *Exophiala* species appear susceptible to amphotericin B, triazole and terbinafine *in vitro*. However, clinical efficacy of antifungals against some strains of *Exophiala* remains controversial as patients have expired despite antifungal therapy [22]. Therefore, correlation of in vitro results and in vivo response due to pharmacokinetics, host factors, onset of infection and therapy remains to be determined. One case report showed *E. dermatitidis* isolate that is resistant to echinocandin class of drugs: caspofungin, microfungin and anidulafungin, but susceptible to azoles and amphotericin B [23]. Another study of 43 isolates of *E. dermatitidis* showed susceptibility to amphotericin B, voriconazole, itraconazole, 5-fluorocytosine and terbinafine. However, this study has demonstrated that amphotericin B has a poor activity against *E. dermatitidis* [24]. Therefore, it is recommended to perform antifungal susceptibility testing immediately when *E. dermatitidis* is suspected as the pathogen in human infections.

**Table 1:** **The result of antifungal susceptibility testing of black fungi assessed by disk diffusion assay**

		**Sample**							
		**Control**				**Black fungi**			
		***C. albicans***		***C. parapsilosis***		**OM2**		**OM4**	
**AMPH**	**ØMean**	14.8	S	12.5	I	9.3	R	12.3	I
	**Range**	13-16		12-13		9-10		12-13	
**CASP5**	**ØMean**	15.5	S	18	S	NA	R	NA	R
	**Range**	14-17		17-19		NA		NA	
**FLUCZ**	**ØMean**	50.8	S	36.5	S	25.3	S	30	S
	**Range**	50-52		35-38		25-26		29-31	
**KETOC**	**ØMean**	53.5	S	52	S	56.3	S	59.7	S
	**Range**	53-54		51-53		56-57		5.9-6	
**ITRAC**	**ØMean**	22	S	18.5	S	25.7	S	26.3	S
	**Range**	21-23		18-19		25-26		26-27	
**VOR. 1**	**ØMean**	52.5	S	45.5	S	52.3	S	53.7	S
	**Range**	51-54		44-47		52-53		53-54	

ØMean = zone diameter in mm; AMPH - Amphotericin B (Resistant: <10 mm; Intermediate: 10 mm - 14 mm; Susceptible: ≥15 mm), CASP5 - Caspofungin (Resistant: ≤12 mm; Intermediate: 13 mm - 15 mm; Susceptible: ≥16 mm), FLUCZ – Fluconazole (Resistant: ≤14 mm; Intermediate: 15 mm - 18 mm; Susceptible: ≥19 mm), KTC – Ketoconazole (Resistant: ≤20 mm; Intermediate: 21 mm - 27 mm; Susceptible: ≥28 mm), ITRAC – Itraconazole (Resistant: <13 mm; Intermediate: 14 mm - 22 mm; Susceptible: ≥23 mm), VOR. 1 – Voriconazole (Resistant: ≤13 mm; Intermediate: 14 mm - 16 mm; Susceptible: ≥17 mm); R-resistant, I-intermediate, S-susceptible.

**Figure 2:**
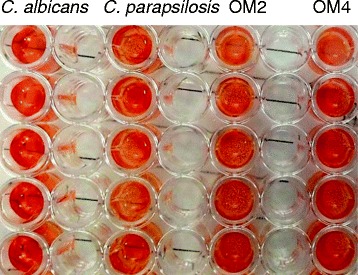
**XTT reduction assay of black fungi after 3-hour incubation.** The darkness of an orange color represented the quantity of biofilm. OM2 and OM4 showed a strong orange color after 3-hour incubation in XTT reduction assay, which was as strong as *C. albicans* and stronger than *C. parapsilosis*. The solution in each well was measured by microtiter plate reader, and the resulted absorbance values were recorded in Table 2.

The results of XTT reduction assay showed that the black fungi, OM2 and OM4 were able to form biofilm (Table 2, Figure 2). Hence, according to the XTT reading, *E. dermatitidis* forms comparable biofilm as good as *C. albicans* SC5314. Biofilm formation is known as an important virulence attribute directly associated with adverse outcome of the *Candida* infections.

**Table 2:** **The result of XTT reduction assay and the hemolysin and proteinase activity of black fungi**

		**XTT**		**Hemolysin**		**Proteinase**	
	**Sample**	**Abs Mean**	**Range**	**Hi Mean**	**Range**	**Pr**_**d**_ **Mean**	**Range**
Control	*C. albicans*	1.92	1.73-2.09	1.73	1.43-2	1.94	1.83-2.07
	*C` parapsilosis*	1.68	1.51-1.94	1.14	1.1-1.13	NA	NA
Black fungi	OM2	2.03	1.8-2.36	2.33	2.22-2.55	NA	NA
	OM4	2.15	1.91-2.43	NA	NA	NA	NA

Abs = absorbance value; Hi = hemolysin index; Prd value = proteinase production index.

**Figure 3:**
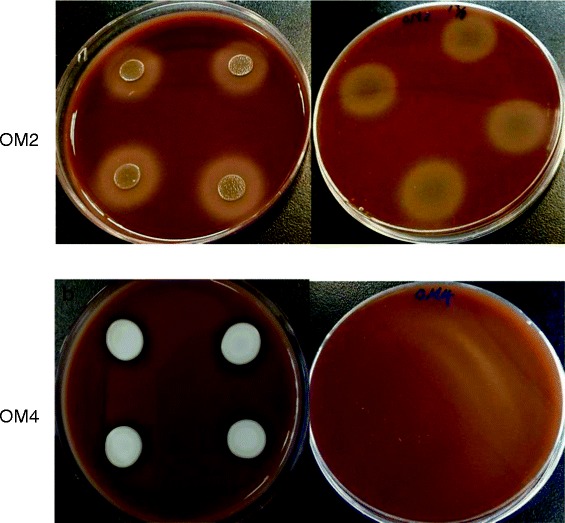
**Photographs showing the hemolysis of sheep blood agar induced by the black fungi.** Picture a and b show the hemolysin activity of OM2 and OM4 respectively. Ten microliters of culture suspension were spotted on the sleep blood agar plate. The plate was observed and the diameter of the halo was measured after 2 days incubation. A distinctive translucent halo around the inoculum site was shown in **a**, which shown positive hemolytic activity of OM2. No halo could be seen in **b**, and therefore OM4 was considered as no hemolytic activity.

It is obvious to see the hemolysis of sheep blood agar induced by *E. dermatitidis* OM2 isolate (Figure 3a). There was a distinctive translucent halo around the inoculum site which showing the positive hemolytic activity. The hemolytic index (Hi) was as high as that of *C. albicans*. On the contrary, *E. dermatitidis* OM4 isolate showed a negative hemolysin activity (Figure 3b). It is an unexpected result as both isolates were recovered from one patient at the same site. Iron plays an important role in the survival of an invasive pathogen like *C. albicans* in the human host. Therefore, invasive pathogens require hemolysin enzymes to extract iron indirectly by lysing iron-containing proteins such as hemoglobin. However, exact clinical implications of these findings remains to be fully elucidated.

## Conclusions

To conclude, we reported the first case of isolation of *E. dermatitidis* species from the human oral cavity. Moreover, we also generated pioneering data on the virulence attributes such as biofilm formation, hemolysin and proteinase assay. Of note, one of these *E. dermatitidis* isolates was resistant to caspofungin and amphotericin B, the two best antifungals available in the market for systemic fungal infections. This finding warranted further clinical studies on this emerging fungal pathogen, particularly among the growing body of the immunocompromised population including patients with NPC.

### Consent

Written informed consent was obtained from the patient for publication of this case report and any accompanying images. A copy of the written consent was available for review by the Editor of this journal.
